# Alignment of Medical and Psychosocial Sectors for Promotion of Tobacco Cessation among Residents of Public Housing: A Feasibility Study

**DOI:** 10.3390/ijerph17217970

**Published:** 2020-10-29

**Authors:** Mandeep S. Jassal, Tracey Oliver-Keyser, Panagis Galiatsatos, Catherine Burdalski, Bonnie Addison, Cassia Lewis-Land, Arlene Butz

**Affiliations:** 1 Department of Pediatrics, Johns Hopkins School of Medicine, Baltimore, MD 21287, USA; baddiso1@jhmi.edu (B.A.); clewis4@jhmi.edu (C.L.-L.); abutz@jhmi.edu (A.B.); 2 Housing Authority of Baltimore City, Baltimore, MD 21287, USA; Tracey.Oliver-Keyser@rsinc.org; 3 Department of Medicine, Johns Hopkins School of Medicine, Baltimore, MD 21287, USA; pgaliat1@jhmi.edu; 4 Johns Hopkins Bayview Medical Center, Department of Pharmacy, Baltimore, MD 21287, USA; cburdal1@jhmi.edu

**Keywords:** public housing, cigarettes, smoking, cessation, psychosocial, social services, environmental tobacco smoke

## Abstract

The inequity in cessation resources is at the forefront of the recently enacted US smoking ban in public housing facilities. This pre-post, non-randomized pilot study assessed the feasibility of a smoking cessation program targeting smokers in Baltimore City public housing. The study implemented a four-phased, 10-week, community-based cessation program using a joint academic–housing partnership that provided on-site cessation pharmacotherapy, behavioral counseling, and psychosocial/legal services. The community-led strategy involved: (1) two-week smoking cessation training for lay health workers; (2) screening and recruitment of smokers by housing authority residential leadership; (3) four-week resident-led cessation using evidenced-based strategies along with wraparound support services; (4) formative evaluation of the intervention’s acceptability and implementation. Thirty participants were recruited of which greater than one-half attended the majority of weekly cessation events. Thirty percent were able to achieve biomarker-proven cessation, as measured by a reduction in exhaled CO levels—a percentage comparable to the reported state quitline 30-day cessation rate. Despite weekly joint community–academic led-education of nicotine replacement therapy (NRT) therapies, only two participants regularly and properly used NRT transdermal patches; <20% of participants used NRT gum correctly at their first follow-up visit. Less than one-half utilized psychosocial and legal services by our community-based organization partners. Post-intervention interviews with participants noted broad approval of the ease in accessibility of the cessation intervention, but more diversification in the timing and personalization of offerings of services would have assisted in greater adoptability and participant retention. Though a reduction in smoking behaviors was not broadly observed, we elucidated modifiable social, educational, and physical features that could enhance the likelihood of smoking cessation among public housing residents.

## 1. Introduction

As of July 2018, the U.S. Department of Housing and Urban Development (HUD) had instituted a smoking ban inside and within 25 feet of public housing agencies (PHAs) [1]. To assist in the implementation of this objective, HUD provided recommendations to PHAs that included partnering with health clinics to provide cessation support, usage of state quitlines, and referrals to healthy housing organizations [2]. This federal decree impacts approximately one-third of low-income public housing residents who smoke tobacco products—the majority of which are conventional cigarettes [3]. Understandably, residents who smoke have expressed frustration since they feel they are being singularly targeted due to their income status and the primary alternative is to smoke outdoors in potentially unsafe settings within public housing (e.g., high-crime zones) [4].

The major modality for promoting the smoking ban policy has been through evidence-based tobacco harm education, cessation pharmacotherapy, and tobacco-centered behavioral counseling [5,6]. However, lower socioeconomic status (SES) populations are significantly less likely to access these evidence-based resources than their resource-replete counterparts [7]. The lack of resource acquisition may be due to fundamental inequities in healthcare structures, whereby lower SES populations are less likely to utilize primary care providers (PCPs) to acquire cessation therapies [8]. Moreover, PCPs may be less likely to provide cessation advice [9]. Lower socioeconomic populations are also more likely to be targeted by the tobacco industry’s marketing campaigns [10]. Another major impediment to the effective delivery of evidence-based cessation interventions for low-income residents of public housing is the potential role of nicotine as an anxiolytic and anti-depressive agent—an important component in its usage where both addiction and socioeconomic stressors (e.g., finances, crime, employment, food security, and housing) often undermine smoking cessation efforts in lower SES settings [11,12,13,14].

Baltimore City is an ideal testing ground for a novel, practical tobacco cessation intervention since it ranks 5th in most public housing units in the USA [15]. The majority of public housing residents are lower-SES African Americans who disproportionately use conventional cigarette products and have a high-prevalence of tobacco-attributed diseases [16,17,18].

Our non-randomized, pre-post pilot utilizes three community-centric core elements: (a) durable and jointly linked community/academic infrastructure systems, (b) recognition and referral to local community-based organizations to address acute psychosocial stressors contributing to smoking behaviors, (c) strong on-site (public housing) residential leadership commitment to cessation improvement. We engaged resident, on-site leaders to be the public face of the intervention since traditional academic staff are unable to promote an intervention with equal potency among residents who identify at geographically granular levels. The resident leaders assisted in the design, implementation, and evaluation of the intervention. This is the first of at least three pilots seeking to refine the development of a public housing-centered smoking cessation program. We hypothesize that the 30-day quit rate will be higher in our joint academic–community led cessation program compared to the Maryland Quitline (the US state-based telephone/online tobacco cessation service in which the study occurred). The state quitline’s 30-day quit rate is reported at 27.9%. However, it must be recognized that this frequently referenced marker is based on a self-report [19]. This 30-day quit rate benchmark is similar to other US quitline programs [20]. We chose using the state quitline as the benchmark since it has been frequently promoted by local PHA sites as a means to enhance cessation [20]. We believe our comprehensive, wrap-around cessation programming that includes on-site smoking harm education, pharmacotherapy, and psychosocial/legal services will be able to enhance the likelihood of achieving high cessation rates

## 2. Materials and Methods

Study design: We conducted a pre-post, non-randomized 10-week pilot study (16 January 2019–27 March 2019) to determine the feasibility of a multi-component cessation program targeting residents in one Baltimore City public housing site. The protocol was approved by the institutional review board at The Johns Hopkins University School of Medicine (IRB00064875).

Study population: Two resident leaders of the housing complex were selected and functioned as research staff. Resident leaders were identified by long-standing, on-site employees of the Office of Resident Services (Housing Authority of Baltimore City). The employees were familiar with both chosen individuals and were strongly recommended for inclusion in the project based on their past involvement in community-based projects. To ensure their position, perception, and relatability to the participants, the two selected adult African American female resident leaders self-identified as being former cigarette smokers and were confirmed by fellow residents as being leaders within the community. Forty participants were screened and enrolled in a week-long recruitment period within the housing community center. We selected participants based on: (a) age ≥18 years; (b) verbally expressing willingness to undertake cessation; (c) primary residence within the public housing site; (d) active smoking status confirmed by exhaled carbon monoxide (CO) > 4 ppm [21]. Participants with exhaled CO that could be ascribed to self-reported marijuana usage were not enrolled. Exclusion criteria included: (a) the use of tobacco cessation pharmacotherapies or services at the time of recruitment, (b) women who were pregnant (or trying to become pregnant) and/or breast-feeding since pharmacotherapy administration was a core component of this intervention, and (c) non-primary residence at the public housing site. Individuals who were excluded were recommended to contact the state quitline or speak to their healthcare provider.

**Figure 1:**
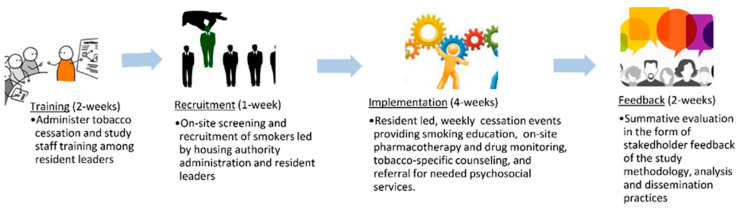
Overview of the four phases of the pilot trial.

Study protocol: The intervention was divided into 4 phases (Figure 1). Resident leaders and academically affiliated research assistants underwent a two-week Johns Hopkins Bayview Medical Center-based continuing medical education (CME)-certified tobacco cessation training program, whose curriculum is tailored to lay healthcare workers. The four themes covered in the workshops included the biological basis of nicotine addiction, pharmacotherapy in tobacco dependence, cognitive management, and social influencers of tobacco usage. The biological basis of nicotine addiction addressed: (a) understanding the construction of cigarettes as a vehicle for enhancing addiction to nicotine, (b) neurobiological changes in nicotine addiction, and (c) role of genetics in addiction. Pharmacology sessions dealt with: (a) medications used for tobacco dependence and (b) usage of controller and reliever medication-based cessation aids when providing treatment for tobacco dependence. Cognitive management discussions involved: (a) role of human behavior towards nicotine addiction, (b) understanding and application of motivation interviewing for tobacco dependence. Social influences of tobacco usage addressed: (a) role of lay and professional healthcare workers in tobacco treatment, (b) the provision of a comfortable approach to enhance cessation of active smokers, and (c) usage of stages of change to enhance likelihood of quitting [22]. Resident leaders and research-affiliated staff underwent pre-post testing of the cessation coursework that included twenty multiple-choice questions that addressed core take-away points from the training sessions.

Behavioral counseling received the largest amount of teaching time in the curriculum. Resident leaders and academic research staff were educated on the application of brief motivation interviewing for tobacco cessation. Time was kept brief for this component based on resident leadership advice that individuals will only wish to spend maximum of 20–30 min on-site for each weekly event. Brief behavioral motivational interviewing applied a modified 5 A’s model: Ask, Advise, Assess, Connect (A3C) [22,23,24]. The “Ask” component consisted of identifying ongoing tobacco usage. The “Advise” component included clear, non-judgemental messaging that portrayed empathy in the struggle to quit; resident leadership was seen as well-positioned to provide messaging given their past usage of cigarettes. The “Assess” component determined their ongoing willingness to quit using stages of change based on the Transtheoretical Mode of Intentional Behavior Change [22]. Lastly, “Connect” further re-emphasized the need to maintain participation or connection with the study intervention. 

Recruitment involved a two-fold strategy using on-site promotion by housing authority-affiliated personnel and direct recruitment efforts by resident leaders. Implementation of the 4-week pilot was undertaken in the housing site’s community center. The individual components of the intervention were delivered weekly using physical stations that were geographically delineated using tables manned by study staff/resident leaders (Supplementary Figure S1). After undergoing verification of study enrollment (Station 1), participants undertook a survey exploring smoking characteristics [Supplementary Figure S2] (Station 2). Exhaled CO levels were acquired using Vitalograph^®^ BreathCO™ (Vitalograph^®^, Lenexa, KS, USA) (Station 3). Weekly supply of nicotine replacement therapy (NRT) in the form of transdermal patches and oral gum/lozenges were disseminated by academic staff, with oversight by a tobacco cessation-specialized pharmacist (Station 4). Over-the-counter NRTs were purchased using non-reimbursed, non-payor mechanisms through grant funding. Selection and general/absolute contraindications of NRTs were reviewed on recruitment and reinforced weekly using questionnaires. Side-effects on therapy were monitored using a 24-h accessible phone line manned by academically trained tobacco cessation specialists. Comprehensive psychosocial service screening and referrals to our Catholic Charities’ Our Daily Bread Employment Center (ODBEC) was undertaken (Station 5). Screening for needs assessment involved application of CDC Community Needs Assessment Training Workbook prioritization grid [15]. If participants listed employment as a stressor “quite a bit” or “far too much”, they would be provided with job-training and placement programs offered remotely at ODBEC headquarters. These ODBEC-based employment-directed programs assist with job searches, interviews, placement, and retention. To complement the pharmacotherapy, resident leaders (with academic oversight) led personalized brief behavioral counseling techniques using the A3C methods taught during the CME specialist training sessions (Station 6) [23,24]. Refreshments were provided as the primary means for reimbursement for study participation. Participants were provided monetary compensation for participation in the study (total $20 provided over four study visits) (Station 7). Maryland Legal Aid (MLA) provided two lawyers offering comprehensive legal services addressing known needs among lower-income communities in Baltimore (Station 8).

Outcome measures: The primary study endpoint was weekly exhaled CO levels. We relied primarily on exhaled CO since it serves as a more objective and reliable biomarker of smoking activity, as opposed to self-report [21,25]. Evaluation of tobacco cessation training among court captains was assessed using a pre/post-course completion questionnaire. Attendance logs were maintained to monitor acceptability of the intervention. Participant fidelity to pharmacotherapy administration techniques occurred through participant demonstration of NRT usage. Logs of participant usage of psychosocial services (including types of needs) were maintained. Formative evaluations used multi-stakeholder interviews, which included the two resident leaders, to: (a) determine the strengths and weaknesses of the study design; (b) systematically detect and monitor unanticipated events; (c) determine the local infrastructure and required funding capacity to sustain the intervention. A brief open ended, semi-structured interview guide with X key domains were developed (Supplementary Table S1). The interviews were conducted by both trained qualitative research staff (CL and BA). Each interview lasted approximately 10 min. Interviews were not digitally recorded and relied on written summations of participant answers by the research team 

Statistical analysis: The primary outcome was an adjusted comparison of the 4-week mean change in exhaled CO (Stata^®^, College Station, TX, USA). Descriptive statistics were generated for medication side effects, weekly follow-up retention, and requested CBO services. Stakeholder interviews were reviewed with study team investigators to identify recurring patterns of beliefs, interactions, behaviors and needs across stakeholder groups. Interview responses were reviewed through an iterative process using principles of qualitative description [26]. Two members of the research team (MJ and AB) reviewed the interview responses and iteratively analyzed them using descriptive analysis methods, to formulate the topical themes to reflect ideas present in the text. Microsoft Excel 2016 was utilized for data management and thematic analysis of the interviews. Interview findings were clustered into future, stakeholder-preferred study design requirements. Given the feasibility objective of this pilot study, as well as inherent study limitations (e.g., budget and single PHA study site), a sample size of thirty was considered a convenient sample size that could accommodate primary and secondary objectives. At that sample size, our study had 80% power to detect a 0.5 proportional mean increase in cessation compared to the state quit line. 

## 3. Results

**Figure 2:**
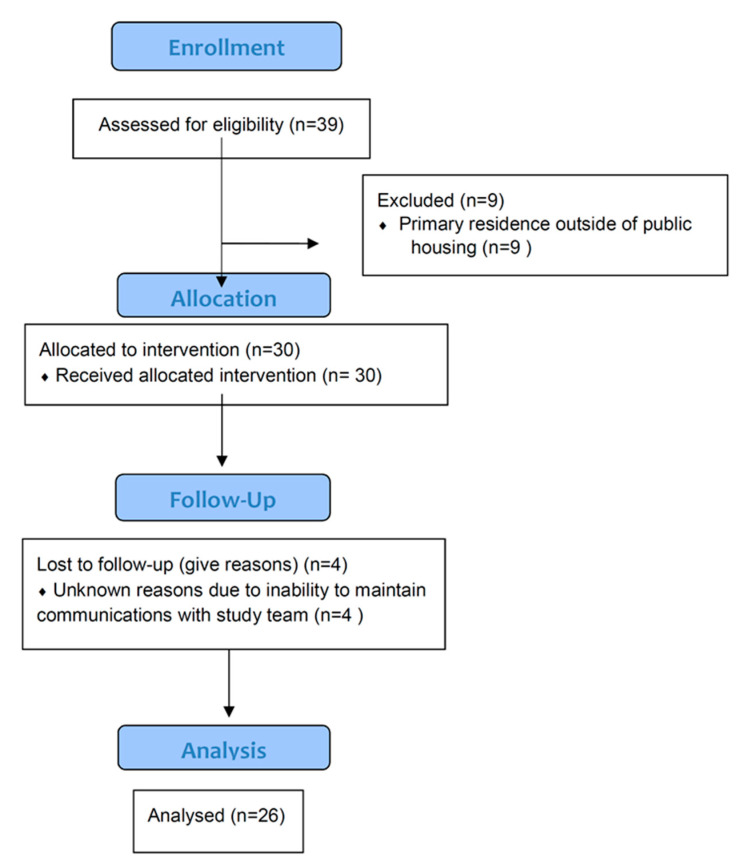
Assessment for eligibility, randomization and follow-up.

All resident leaders and academically affiliated research assistants demonstrated complete knowledge, with scores of 100%, on post-training examinations. Thirty-nine participants were recruited in two hours, but nine were excluded due to their residence outside of the public housing unit (Figure 2). Excluded participants reported that word-of-mouth conversations from public housing residents resulted in their attendance at the recruitment event. Among the 30 participants who were recruited, four participants were lost to follow-up and could not be contacted by phone to acquire the underlying reasons. Twenty-six participants had attended at least two study visits, including the final visit. Fourteen participants had attended at least three visits of which 10 participated in all sessions. Among the 26 recruited, 58% were female, African American and between the ages of 41–60 years of age (Table 1). Most participants initiated cigarette smoking in adolescence and did not engage in any cessation activity in the 12 months prior to the intervention. 

Exhaled CO levels were slightly lower, though not meaningfully different among participants before or after the intervention when accounting for compliance in usage of NRT products and usage of psychosocial/legal services (mean pre-CO level = 10.08; mean post-CO level = 9.08) (Supplementary Figure S2, Table S2). Individual analyses showed that 30.7% (eight out of 26 participants) achieved cessation based on exhaled CO (Table 2). Only two people consistently reported using transdermal NRT patches as a daily controller therapy. All participants reported use of NRT gum or lozenges multiple times per day. Despite NRT gum being preferable, <20% of participants were using the gum correctly at their first follow-up visit. Patches were the only product for which side-effects were reported, including jitteriness, nausea, itchiness, and difficulty with maintaining placement on the skin. Among participants claiming adherence to NRT therapies, side-effects and/or difficulty with usage of NRT products were addressed by our on-site tobacco-treatment pharmacist and no change in the weekly regimens were required. Adherence to NRTs (oral and/or transdermal) did not correlate with biomarker-established cessation (Table 2). The magnitude of cigarette usage in the 30 days before and after the initiation of the intervention generally correlated with biomarker usage.

Tobacco-centered behavioral counseling was not utilized by every participant at each weekly event. Surveys of the most frequently reported psychosocial needs revealed, in order: employment, housing security, substance usage treatment, mental health and neighborhood crime (Supplementary Table S2). Greater than 80% of participants commented on the need for greater offering of psychosocial services, despite only 46% (*n* = 12) of individuals utilizing the offerings of our community partner organizations. Twelve participants did avail of on-site Maryland Legal Aid services for which all but one addressed criminal expungement issues.

Post-intervention interviews of participants noted that most preferred the accessibility of the cessation intervention (Table 3). They also preferred the perception of our team, comprised of local resident leadership, displaying empathy by focusing on issues of self-betterment. Commonly reported programmatic issues requiring improvement included more frequent offerings of the intervention, as opposed to solely weekly evening events. More staffing and efficient time usage was frequently cited as areas to improve in future pilot efforts.

## 4. Discussion

The pilot study demonstrated the strengths and weaknesses of a community-centered, joint academic-residential smoking cessation program targeting low-income public housing residents. The program was constructed and marketed during recruitment as a means to address the recent US public housing smoking ban. Despite the interest and general positive resident feedback towards the program, we were unable to reduce exhaled CO levels. We were able to approximate the success rates reported by the state quitline 30-day cessation benchmark of 27.9% [19,20]. Vast difficulties in the correct provision of NRTs was observed with notable poor adherence to daily transdermal patches and incorrect usage of gum/lozenge products. Behavioral counseling delivered by residential leadership, with academic oversight, was non-optimally utilized. A minority of residents solely utilized on-site legal aid services, yet none utilized off-site, more comprehensive services by our off-site community partners.

Several limitations existed in the multi-phased feasibility study. Our hypothesis was based on exceeding the 30-day quit rate of our state quitline (27.9%), but that benchmark is based on self-report which may be prone to reporting bias [19,20]. We relied solely on biomarker-proven cessation and thus our results may not be comparable. Another major limitation that will be rectified in upcoming trials was the lack of research staff and direct participant feedback on the application and benefit of the brief behavioral counseling techniques. Our usage of a brief (approximately 5 min) “A3C” form of motivation interviewing to promote cessation may not have been the ideal intervention for a population with a variety or diverse psychological and socioeconomic stressors [23,24]. The cessation training was isolated to two weeks for the resident leaders, which may have been insufficient for the education of non-healthcare or research-oriented affiliates of the program. Characterizing the sufficiency of the training was not optimal since it relied solely on pre- and post-test evaluations, as opposed to observing or demonstrating successful implementation of cessation themes. Another notable limitation was the shortened timespan of the implementation phase of four weeks, which can be ascribed to budgetary constraints. Four weeks would likely be insufficient for assessing the maximal benefit of cessation pharmacotherapy that may take multiple months to achieve optimal effect [27]. Our sample size for this study was purposely kept limited based on our study objectives formulated primarily for feasibility. Future work is planned to enhance the sample size to power a study, including the inclusion of multiple PHA sites, to further assess both feasibility and efficacy of cessation efforts. We also did not adequately capture the magnitude of cigarette usage reduction since we relied on broad ranges of reported cigarette usage, as opposed to exact values. In general, our trends correlated well with exhaled CO levels and thus we primarily reported on the more objective, quantitative value. Moreover, we did not sufficiently document demonstration and education of appropriate NRT usage of each participants at the weekly study visits—a limitation that is relevant since the minority of our cohort were noted to properly use NRTs. Further iterations will be improved by the inclusion of a station dedicated solely to the repeated demonstration of appropriate usage of NRTs. We also did not capture time spent at each station by each participant, which would have served as a helpful observation allowing us to refine the marketing and utilization of core components of the intervention, including the utilization of psychosocial services. The inadequate utilization at each station may have additionally been informed by querying participants after each weekly session on the reasons why the stations were not perceived beneficial, despite their perceived relevance in addressing the nicotine and multiple psychosocial stressors attribute to cigarette usage. Lastly, formative evaluations did not specifically query the viewpoints of participants on the usage of solely female resident leaders. Future work may potentially benefit from the inclusion of more men as residential leaders since our solely female resident-led pilot may not have been as relatable for male smokers.

This project represents our first of multiple planned pilot trials to enhance smoking cessation among public housing residents. The next planned iteration is based on the analyses and feedback derived from this initial pilot. Features that are being prioritized include more repetitive and personalized education of NRT products, along with diversification of tobacco pharmacotherapies, with continued oversight by tobacco-treatment pharmacist. Purchasing of tobacco pharmacotherapy for on-site dissemination using insurance-based modalities is being investigated given the high-cost, potentially unsustainable approach of using grant funding for NRT products. Moreover, the concern for inadequate numbers of research staff to assist in the efficiency of the program implementation will be addressed by recruiting more residential leaders as research staff. The next iteration will use a more personalized marketing of psychosocial/legal services by advertising individually relevant services that each community-based organization offers. Along with promotion, future work will benefit from inclusion of a transport system to enhance access to off-site partners.

## 5. Conclusions

Our feasibility study shows the general positive receptivity, as well as significant limitations in our public housing-centered smoking cessation intervention. The observed weaknesses are surmountable and, if successfully addressed, a highly needed program can be provided to address the disproportionate usage of smoking and subsequent health inequities in low-income residential settings. Ultimately, the work serves as the foundation to build and further improve health equity promoting projects in a susceptible and receptive population of residents in US public housing.

## Figures and Tables

**Table 1:** Baseline participant demographic, smoking and psychosocial characteristics (*n* = 26).

Characteristics	N
Age	
20–40	6
41–60	15
>60	5
Gender	
Male	11
Female	15
Number of cigarettes smoked per day	
1×/day	2
2–5×/day	4
6–10×/day	11
11–20×/day	3
>20×/day	6
Age of initiation of smoking	
≤21 years old	21
>21 years old	3
Not reported	2
Attempted cessation at least once in past year	
No	19
Yes	7

**Table 2:** Breakdown of individual exhaled CO levels (week 0 and week 4), achievement of biomarker-defined cessation, NRT compliance at 4 weeks (transdermal and oral options) and magnitude of cigarette consumption (week 0 and week 4). Biomarker-based cessation was defined as an exhaled CO level ≤ 4 ppm [14].

Participant	Exhaled CO (Week 0)	Exhaled CO (Week 4)	Achievement of Cessation	Week 4 NRT Transdermal Compliance	Week 4 NRT Oral Compliance	Average Cigarette Smoked 30 Days Prior (Week 0)	Average Cigarette Smoked 30 Days Prior (Week 4)
1	8	1	YES	N	Y	6 to 10 per day	1 or 2 days
2	8	5	NO	N	Y	More than 20 per day	10 to 19 days
3	9	10	NO	N	N	11 to 20 per day	10 to 19 days
4	11	14	NO	N	N	11 to 20 per day	28 or more days
5	19	16	NO	N	N	6 to 10 per day	6 to 9 days
6	27	1	YES	N	Y	6 to 10 per day	3 to 5 days
7	5	4	YES	N	Y	6 to 10 per day	28 or more days
8	7	8	NO	N	Y	6 to 10 per day	28 or more days
9	5	12	NO	Y	Y	1 per day	10 to 19 days
10	10	1	YES	N	Y	More than 20 per day	3 to 5 days
11	6	14	NO	N	N	More than 20 per day	28 or more days
12	20	27	NO	N	Y	6 to 10 per day	10 to 19 days
13	10	9	NO	N	N	11 to 20 per day	28 or more days
14	5	2	YES	N	N	More than 20 per day	3 to 5 days
15	9	2	YES	N	Y	2 to 5 per day	1 or 2 days
16	11	13	NO	N	Y	2 to 5 per day	28 or more days
17	14	22	NO	N	N	6 to 10 per day	28 or more days
18	6	10	NO	N	N	6 to 10 per day	3 to 5 days
19	17	6	NO	N	Y	28 or more days	6 to 9 days
20	13	18	NO	N	N	6 to 10 per day	28 or more days
21	9	11	NO	N	N	6 to 10 per day	10 to 19 days
22	4	1	YES	N	N	2 to 5 per day	1 or 2 days
23	7	6	NO	N	Y	More than 20 per day	28 or more days
24	13	17	NO	N	N	2 to 5 per day	28 or more days
25	5	1	YES	N	N	2 to 5 per day	3 to 5 days
26	6	5	NO	Y	N	6 to 10 per day	28 or more days

**Table 3:** Common reported concerns by participants regarding the intervention.

3 Areas That Were Well-Received	8 Areas Requiring Improvement
Better way to conceive and adopt actions leading to smoking cessation	Need more on-site psychosocial services
Ease in acquisition and education of NRTs	Need more offered times for implementation of the intervention (do not prefer just once weekly events)
Demonstration of interest by our team in the resident’s self-betterment	Shorten the time required for surveys and other academic-related endpoints
	More staff to enhance participant flow through the multi-component intervention
	More education material (different media) addressing smoking cessation
	Limited time of intervention with no stated date for re-implementation of the program
	Provide monetary reimbursement only at the final session
	Non-optimal advertisement of the pilot program *(requires more than limited distribution of flyers)*

## Supplementary Materials

The following are available online at https://www.mdpi.com/1660-4601/17/21/7970/s1. Supplementary Figure S1. Physical layout of the weekly intervention within the community center. Participants walk progressively from stations 1–8 in the upstairs and downstairs areas of the community center. Supplementary Figure S2. Surveys used during baseline and follow-up visits. Material was adapted from CDC and PROMIS^®^ Smoking Initiative survey tool measures. Supplementary Figure S3. Pre-post exhaled CO levels. Supplementary Table S1. Key domains and sample question topics. Supplementary Table S2. Magnitude of psychosocial and legal stressors ranked reported by participants, ranked by order of need. Each stressor was ranked based on a scale of 1-3, with a score of 1 indicating low need and 3 representing the highest need.

## References

[1] National Center for Housing Management HUD Mandates Smoke-Free Public Housing. http://www.nchm.org/Resources/Compliance-Corner/Review/ArticleId/163/Public-Housing-Goes-Smoke-Free.

[2] Department of Housing and Urban Development Implementing HUD’s Smoke-Free Policy in Public Housing. https://www.hud.gov/sites/documents/SMOKEFREE_GUIDEBK.PDF.

[3] Helms V.E., King B.A., Ashley P.J. (2017). Cigarette smoking and adverse health outcomes among adults receiving federal housing assistance. Prev. Med..

[4] The Baltimore Sun (Reporters: Campbell C, Prundente T) HUD to Ban Smoking in All Federally Subsidized Public Housing. https://www.baltimoresun.com/maryland/bs-md-hud-smoking-ban-20161130-story.html.

[5] Tobacco Use and Dependence Guideline Panel (2008). Treating Tobacco Use and Dependence: 2008 Update.

[6] (2017). Final Recommendation Statement: Tobacco Smoking Cessation in Adults, Including Pregnant Women: Behavioral and Pharmacotherapy Interventions. https://www.uspreventiveservicestaskforce.org/Page/Document/RecommendationStatementFinal/tobacco-use-in-adults-and-pregnant-women-counseling-and-interventions.

[7] Garrett B.E., Dube S.R., Babb S., McAfee T. (2015). Addressing the Social Determinants of Health to Reduce Tobacco-Related Disparities. Nicotine Tob. Res..

[8] Bailey S.R., Heintzman J., Jacob R.L., Puro J., Marino M. (2018). Disparities in Smoking Cessation Assistance in US Primary Care Clinics. Am. J. Public Health.

[9] Haas J.S., Linder J.A., Park E.R., Gonzalez I., Rigotti N.A., Klinger E.V., Kontos E.Z., Zaslavsky A.M., Brawarsky P., Marinacci L.X. (2015). Proactive tobacco cessation outreach to smokers of low socioeconomic status: A randomized clinical trial. JAMA Intern Med..

[10] Moran M.B., Heley K., Pierce J.P., Niaura R., Strong D., Abrams D. (2019). Ethnic and Socioeconomic Disparities in Recalled Exposure to and Self-Reported Impact of Tobacco Marketing and Promotions. Health Commun..

[11] Picciotto M.R., Brunzell D.H., Caldarone B.J. (2002). Effect of nicotine and nicotinic receptors on anxiety and depression. Neuroreport.

[12] Castro Y., Costello T.J., Correa-Fernández V., Heppner W.L., Reitzel L.R., Cofta-Woerpel L., Mazas C.A., Cinciripini P.M., Wetter D.W. (2011). Differential effects of depression on smoking cessation in a diverse sample of smokers in treatment. Am. J. Prev. Med..

[13] Levinson A.H. (2017). Where the U.S. tobacco epidemic still rages: Most remaining smokers have lower socioeconomic status. J. Health Care Poor Underserved.

[14] Slopen N., Dutra L.M., Williams D.R., Mujahid M.S., Lewis T.T., Bennett G.G., Ryff C.D., Albert M.A. (2012). Psychosocial stressors and cigarette smoking among African American adults in midlife. Nicotine Tob. Res..

[15] Housing Authority of Baltimore City 4CS of HABC—Mission, Vision and Goals. https://www.habc.org/habc-information/about-us/4cs-of-habc-mission-vision-and-goals/.

[16] Spencer M., Petteway R., Bacetti L., Barbot O., Healthy Baltimore 2015: A city where all residents realize their full health potential. Baltimore City Health Department, May 2011 http://health.baltimorecity.gov/sites/default/files/HealthyBaltimore2015_Final_Web.pdf.

[17] United States Census Bureau QuickFacts Baltimore City, Maryland. https://www.census.gov/quickfacts/fact/table/baltimorecitymaryland/PST045217.

[18] Kegler M.C., Lebow-Skelley E., Lea J., Lefevre A.M., Diggs P., Herndon S., Haardörfer R. (2018). Developing Smoke-Free Policies in Public Housing: Perspectives from Early Adopters in 2 Southern States. Prev. Chronic Dis..

[19] Maryland Department of Health Maryland Tobacco Control and Prevention Program Interim Evaluation. 2018. https://phpa.health.maryland.gov/ohpetup/Documents/Full%20Report%20-%20Interim%20Evaluation%20Findings%206.13.18.pdf.

[20] Vickerman K.A., Schauer G.L., Malarcher A.M., Zhang L., Mowery P., Nash C.M. (2015). Quitline Use and Outcomes among Callers with and without Mental Health Conditions: A 7-Month Follow-Up Evaluation in Three States. Biomed. Res. Int..

[21] Cropsey K.L., Trent L.R., Clark C.B., Stevens E.N., Lahti A.C., Hendricks P.S. (2014). How low should you go? Determining the optimal cutoff for exhaled carbon monoxide to confirm smoking abstinence when using cotinine as reference. Nicotine Tob. Res..

[22] Prochaska J.O., Velicer W.F. (1997). The transtheoretical model of health behavior change. Am. J. Health Promot..

[23] Vidrine J.I., Shete S., Cao Y., Greisinger A., Harmonson P., Sharp B., Miles L., Zbikowski S.M., Wetter D.W. (2013). Ask-Advise-Connect: A new approach to smoking treatment delivery in health care settings. JAMA Intern Med..

[24] MDQuit.org Brief Interventions. https://mdquit.org/cessation-programs/brief-interventions.

[25] Sandberg A., Sköld C.M., Grunewald J., Eklund A., Wheelock Å.M. (2011). Assessing recent smoking status by measuring exhaled carbon monoxide levels. PLoS ONE.

[26] Sandelowski M. (2000). Whatever Happened to Qualitative Description?. Res. Nurs. Health.

[27] Leone F.T., Baldassarri S.R., Galiatsatos P., Schnoll R. (2018). Nicotine Dependence: Future Opportunities and Emerging Clinical Challenges. Ann. Am. Thorac. Soc..

