# Supplementary material for: Alignment of Medical and Psychosocial Sectors for Promotion of Tobacco Cessation among Residents of Public Housing: A Feasibility Study
Source: Int J Environ Res Public Health. 2020 Oct 29;17(21):7970. doi: 10.3390/ijerph17217970 (PMC7663453; doi:10.3390/ijerph17217970)

Supplementary Table S1. Key domains and sample question topics.

**Primary psychosocial stressors**
• Primary psychosocial or financial issues that potentially link with cigarette usage
**Opinions on the current intervention**
• Resident leadership and incorporation into the intervention
• Pharmacotherapy selection, education and delivery
• Site, timing and frequency of the intervention
• Research staff and participant relationships
• Contribution, involvement and participation of community partners (Catholic Charities and Our Daily Bread)
• Length of time and ease of completion of surveys
• Marketing of the intervention

Supplementary Table S2. Magnitude of psychosocial and legal stressors ranked reported by participants, ranked by order of need. Each stressor was ranked based on a scale of 1-3, with a score of 1 indicating low need and 3 representing the highest need.

Psychosocial and legal stressors	Ranking
Employment	28
Housing security	22
Substance abuse	21
Mental/behavioral health	20
Crime/neighborhood safety	16
Hunger or lack of food	15
Chronic disease	15
Social security SSI	15
General health	8
Dental or teeth	8
Debt	8
Felony/misdemeanor expungement	3
Child education	3
Divorce or separation	2
Child custody	0
Adult education	0

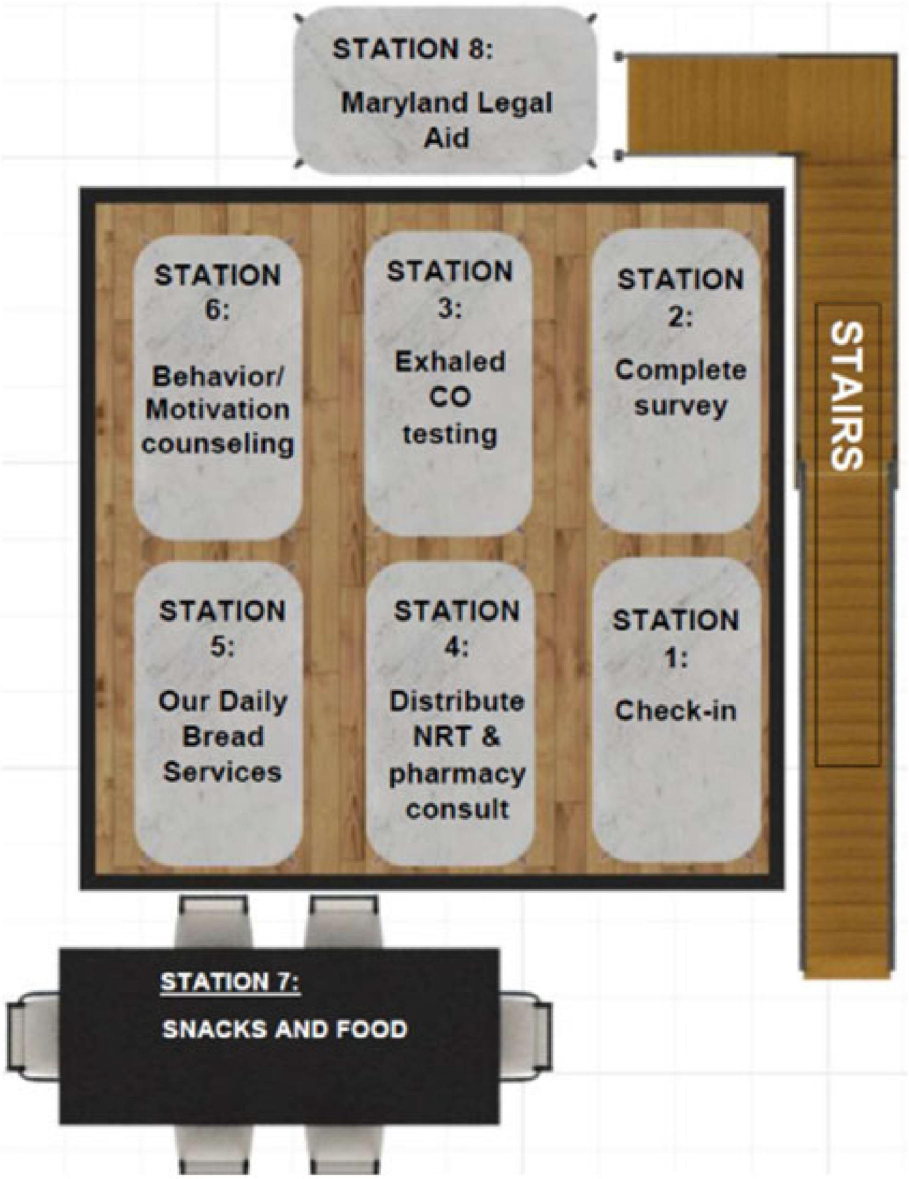

Supplementary Figure S1. Physical layout of the weekly intervention within the community center. Participants walk progressively from stations 1–8 in the upstairs and downstairs areas of the community center.

Supplementary Figure S2._Surveys used during baseline and follow-up visits. Material was adapted from CDC and PROMIS® Smoking Initiative survey tool measures.

COMPANION QUESTIONS (USED ONLY DURING BASELINE VISIT)

How long have you been smoking cigarettes?

- ☐ Less than 1 year
- ☐ 1-2 years
- ☐ 3-5 years
- ☐ 6-9 years
- ☐ 10 years or more

How old were you when you first started smoking?

______ ______

During the past 30 days, on how many days did you smoke cigarettes?

- ☐ 0 days
- ☐ 1 or 2 days
- ☐ 3 to 5 days
- ☐ 6 to 9 days
- ☐ 10 to 19 days
- ☐ 20 to 27 days
- ☐ 28 or more days

During the past 30 days, on average, how many cigarettes per day have you smoked?

- ☐ I did not smoke during the past 30 days
- ☐ Less than 1 per day
- ☐ 1 per day
- ☐ 2 to 5 per day
- ☐ 6 to 10 per day
- ☐ 11 to 20 per day
- ☐ More than 20 per day

After I wake up I typically smoke my first cigarette of the day…

- ☐ Within 5 minutes after waking
- ☐ 6 to 30 minutes after waking
- ☐ 31-60 minutes after waking
- ☐ longer than 60 minutes after waking

What brand of cigarettes do you usually smoke?

Enter brand name:__________

During the past 12 months, have you stopped smoking for 24 hours or more because you were trying to quit?

☐ Yes
☐ No

If you have tried to quit tobacco in the past, what helped you (may select more than one)?

☐ Nicotine Patch
☐ Nicotine Gum
☐ Nicotine Nasal Spray
☐ Zyban or Wellbutrin
☐ Chantix
☐ "Cold Turkey"
☐ Counseling
☐ Nothing helped
☐ Other: ____________________

Do you live or take care of a child inside your home?

☐ Yes
☐ No → If no, then move to the next section

During the last 7 days, in which of the following places has your child been exposed to secondhand smoke?

☐ At home
☐ At another parent's house (e.g., shared custody)
☐ In my car
☐ In someone else's car or another vehicle
☐ In an outdoor public place
☐ At a relative's house
☐ At a friend's house
☐ At daycare or school

☐ Other (please specify): ______________
☐ My child has not been exposed to secondhand smoke in the last 7 days

Please tell me which best describes how cigarette smoking is handled at your home (home includes porches and balconies)

☐ No one is allowed to smoke anywhere
☐ Smoking is permitted in some places or at some times
☐ Smoking is permitted anywhere

Smoking: Nicotine Dependence for Daily and Nondaily Smokers – Short Form 4a

Please respond to each question or statement by marking one box per row.

		Never	Rarely	Sometimes	Often	Always
SMKNDEP01	When I haven't been able to smoke for a few hours, the craving gets intolerable…...	☐ 1	☐ 2	☐ 3	☐ 4	☐ 5
SMKNDEP02	I find myself reaching for cigarettes without thinking about it…………………	☐ 1	☐ 2	☐ 3	☐ 4	☐ 5
SMKNDEP03	I drop everything to go out and buy cigarettes………………………………….	☐ 1	☐ 2	☐ 3	☐ 4	☐ 5
SMKNDEP04	I smoke more before going into a situation where smoking is not allowed……………	☐ 1	☐ 2	☐ 3	☐ 4	☐ 5

Smoking: Coping Expectancies for Daily and Nondaily Smokers – Short Form 4a

Please respond to each question or statement by marking one box per row.

		Never	Rarely	Sometimes	Often	Always
SMKCOP02	When I'm angry, a cigarette can calm me down……………………………………...	☐ 1	☐ 2	☐ 3	☐ 4	☐ 5
SMKCOP04	I am tempted to smoke when I feel depressed…………………………………	☐ 1	☐ 2	☐ 3	☐ 4	☐ 5
		**Not at all**	**A little bit**	**Somewhat**	**Quite a bit**	**Very much**
SMKCOP01	I rely on smoking to deal with stress……..	☐ 1	☐ 2	☐ 3	☐ 4	☐ 5
SMKCOP03	Smoking allows me to take a break from my problems for a few minutes…………..	☐ 1	☐ 2	☐ 3	☐ 4	☐ 5

Smoking: Negative Health Expectancies for Daily and Nondaily Smokers – Short Form 6a

Please respond to each question or statement by marking one box per row.

		Not at all	A little bit	Somewhat	Quite a bit	Very much
SMKHLTH01	Smoking is taking years off my life………..	☐ 1	☐ 2	☐ 3	☐ 4	☐ 5
SMKHLTH02	Smoking makes me worry about getting heart troubles……………………………….	☐ 1	☐ 2	☐ 3	☐ 4	☐ 5
SMKHLTH03	Smoking causes me to get tired easily……..	☐ 1	☐ 2	☐ 3	☐ 4	☐ 5
SMKHLTH04	Smoking makes me short of breath………...	☐ 1	☐ 2	☐ 3	☐ 4	☐ 5
SMKHLTH05	Smoking irritates my mouth and throat…….	☐ 1	☐ 2	☐ 3	☐ 4	☐ 5
SMKHLTH06	I worry that smoking will lower my quality of life……………………………………….	☐ 1	☐ 2	☐ 3	☐ 4	☐ 5

Smoking: Negative Psychosocial Expectancies for Daily and Nondaily Smokers – Short Form 6a

Please respond to each question or statement by marking one box per row.

		Not at all	A little bit	Somewhat	Quite a bit	Very much
SMKPSY01	If I quit smoking I will be more in control of my life…………………………………...	☐ 1	☐ 2	☐ 3	☐ 4	☐ 5
SMKPSY02	If I quit smoking my friends will respect me more………………………………………..	☐ 1	☐ 2	☐ 3	☐ 4	☐ 5
SMKPSY03	My need for cigarettes makes me feel disappointed in myself……………………..	☐ 1	☐ 2	☐ 3	☐ 4	☐ 5
SMKPSY04	My smoking makes me feel less attractive...	☐ 1	☐ 2	☐ 3	☐ 4	☐ 5
SMKPSY05	People think less of me if they see me smoking…………………………………….	☐ 1	☐ 2	☐ 3	☐ 4	☐ 5
SMKPSY06	My cigarette smoking bothers others………	☐ 1	☐ 2	☐ 3	☐ 4	☐ 5

PROMIS Short Form v1.0 – Smoking: Social Motivations for Daily and Nondaily Smokers 4a

Smoking: Social Motivations for Daily and Nondaily Smokers – Short Form 4a

Please respond to each question or statement by marking one box per row.

		Not at all	A little bit	Somewhat	Quite a bit	Very much
SMKSOC01	Smoking makes me feel better in social situations………………………………...	☐ 1	☐ 2	☐ 3	☐ 4	☐ 5
SMKSOC02	Smoking helps me feel more relaxed when I'm with other people…………….	☐ 1	☐ 2	☐ 3	☐ 4	☐ 5

SMKSOC03	I feel like part of a group when I'm around other smokers………..………….	☐ 1	☐ 2	☐ 3	☐ 4	☐ 5
SMKSOC04	I enjoy the social aspect of smoking with other smokers…………………………...	☐ 1	☐ 2	☐ 3	☐ 4	☐ 5

Supplementary Figure 3. Pre-post exhaled CO levels.

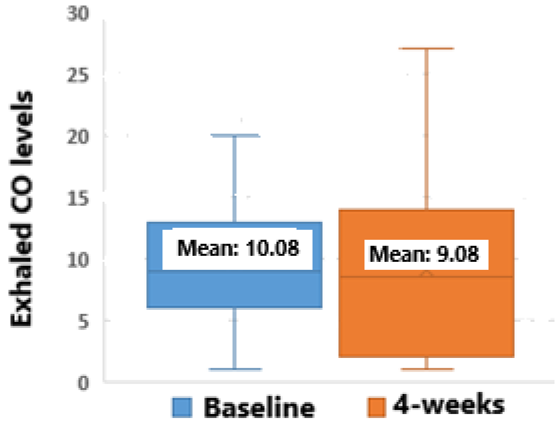

Supplement: Supplementary file 1 [file ijerph-17-07970-s001.pdf]
